# Dietary Pomegranate Pulp: Effect on Ewe Milk Quality during Late Lactation

**DOI:** 10.3390/ani9050283

**Published:** 2019-05-27

**Authors:** Bernardo Valenti, Giuseppe Luciano, Luciano Morbidini, Umberto Rossetti, Michela Codini, Marcella Avondo, Alessandro Priolo, Marco Bella, Antonio Natalello, Mariano Pauselli

**Affiliations:** 1 Dipartimento di Scienze Agrarie, Alimentari ed Ambientali, University of Perugia, Borgo XX Giugno 74, 06121 Perugia, Italy; luciano.morbidini@unipg.it (L.M.); umberto.rossetti@outlook.it (U.R.); mariano.pauselli@unipg.it (M.P.); 2 Dipartimento di Agricoltura, Alimentazione e Ambiente, University of Catania, Via Valdisavoia 5, 95123 Catania, Italy; giuseppe.luciano@unict.it (G.L.); mavondo@unict.it (M.A.); alessandro.priolo@unict.it (A.P.); bellamarco@yahoo.it (M.B.); antonio.natalello@unict.it (A.N.); 3 Dipartimento di Scienze Farmaceutiche, University of Perugia, Via Ariodante Fabretti 48, 06123 Perugia, Italy; michela.codini@unipg.it

**Keywords:** milk, fatty acids, antioxidant capacity, pomegranate, late lactation

## Abstract

**Simple Summary:**

The agro-industrial by-products from the pomegranate juice industry are rich in bioactive compounds. The inclusion of such by-products in the diet of lactating ruminants could improve the health benefits of milk and its technological properties, such as the cheese-making, especially in certain conditions. For example, during late lactation when the quality of milk naturally declines or during the dry season when the availability of good-quality pasture is limited. In addition, recycling agro-industrial by-products that are not edible by humans as an ingredient of the animal diet could reduce the feed costs and the feed-to-food competition in livestock production.

**Abstract:**

Pomegranate pulp, a by-product of the pomegranate juice industry, contains a remarkable quantity of bioactive compounds that can favorably affect ruminant metabolism and milk quality. The present paper investigated the effect of dietary pomegranate pulp on milk yield and quality during late lactation in grazing ewes. Twenty Comisana ewes (150 ± 10 days in milk) were subdivided into control (CTRL) and pomegranate (PP) groups. The CTRL group received a corn-barley based concentrate, while the PP group received a concentrate containing 64.8% pomegranate pulp. Dietary treatment did not affect milk yield. CTRL milk had a greater percentage of *β*-casein and total casein, while *α*_s1_-casein percentage tended to be greater in the PP group. The PP milk showed a lower percentage of 14:0, 16:0, but a greater percentage of vaccenic, rumenic, and *α*-linolenic acid. Punicic acid was detected only in the PP milk. Total antioxidant capacity (ORAC) was greater in the CTRL milk as compared with the hydrophilic ORAC. Dietary pomegranate pulp increased milk health quality with no detrimental effects on milk yield. Therefore, pomegranate pulp could represent a strategy for improving milk quality and reducing feeding cost during a less profitable phases such as late lactation. Also, dietary pomegranate pulp, as an alternative to traditional feedstuffs, may lower feed-to-food competition in livestock production.

## 1. Introduction

Pomegranate is one of the most ancient cultivated trees traditionally used in folk medicine in the Middle East. Scientific investigations have provided evidence that the health properties of pomegranate mainly depend on the phytochemicals present in both the edible and non-edible portions of the fruit [1]. The juice contains a valuable quantity of anthocyanins, flavonoids, and hydroxyl benzoic acids [2] while the peel is rich in hydrolysable tannins, among which punicalagin is the dominant and peculiar compound [3]. Also, pomegranate seeds are a source of polyunsaturated fatty acids (PUFA), among which punicic acid (PA; *cis*-9 *trans*-11 c*is*-13 18:3) has been proven to be beneficial for human health [4].

Pomegranate pulp is one of the by-products produced during pomegranate juice extraction and consists of variable proportions of peel, seeds, and residual pulp. The content of fiber, crude protein, and fat makes this by-product a suitable feed to be included at a high percentage in the diet of ruminants. Therefore, the recycling of pomegranate pulp as an ingredient of ruminant feeds could contribute to the reduction in the feed-to-food competition of animal production by replacing potentially human-edible feeds such as cereals and, at the same time, could represent a viable strategy to reduce the disposal costs for the industry. In addition, the residual bioactive compounds could improve rumen protein metabolism or impair the biohydrogenation of dietary PUFA [5]. Also, most of these compounds are potent free radical scavengers that could favorably affect the antioxidant status of the animal [6] and some of them can be absorbed and accumulated in milk and meat, improving their nutritive value and shelf life [5]. These properties might be of particular interest in some areas, such as the Mediterranean, to attenuate the impact of the seasonal availability of good quality pasture on the yield and composition of sheep milk, or during late lactation when milk yield and quality physiologically degrade.

Agro-industrial by-products of pomegranate have been investigated to evaluate the effect on ruminal fermentation, performance, and product quality in large [7] and small ruminants [8,9]. However, recent research has only focused on the effect of pomegranate by-products obtained from single parts of the fruit. In contrast, the whole pomegranate pulp, where all the above-described phytochemicals are present at once, has been scarcely investigated. Therefore, only the effects of feeding PP silage has been investigated in cow milk and lamb meat production [10,11]. In addition, to the best of our knowledge, pomegranate pulp has not been previously tested on dairy ewes. In the light of above, the aim of this study was to assess the effect of dietary pomegranate pulp on the yield and quality of milk from grazing ewes in advanced stage of lactation.

## 2. Materials and Methods

### 2.1. Dried Pomegranate Pulp

The fresh pomegranate pulp was obtained from a local juice factory. At the factory, the pomegranate fruits (Wonderful variety) were halved and squeezed mechanically. Approximately 300 kg of the residual part containing peels, seeds, membranes, and portion of arils was collected and dried in a ventilated oven at 40 °C until a constant weight. The dried pomegranate pulp was minced, and a representative sample was collected and analyzed for the chemical composition in order to establish the level of inclusion in the experimental concentrate.

### 2.2. Animals and Diet

The present study was conducted in the facilities of the University of Perugia, from April 2016 to June 2016 and the experimental plan was approved by the Universities of Perugia and Catania. Twenty multiparous Comisana lactating ewes at 150 ± 10 DIM were randomly assigned to two groups (n = 10), namely control (CTRL) and pomegranate pulp (PP), balanced for parity, current milk yield (486 vs. 501 g/day), and BW (64.7 vs. 66.2 kg). During the 21-day experimental period, all the ewes grazed together on a native pasture (*Medicago arabica* (L.) *Hudson*, 35%; *Bellis perennis* L., 25%; *Bromus hordeaceus* L., 10%; *Dactylis glomerata* L., 10%; *Brachypodium rupestre* (Host) R. et S., 5%; *Hordeum murinum* L., 5%; *Plantago lanceolata* L., 5%; other 5%) 6 h per day using a rotational grazing system. Each pasture parcel was grazed on two consecutive days by moving electrified fence in order to provide approximately 40 m^2^/ewe/day, while water was always available. After grazing, the animals were kept in sawdust bedded pens and were offered 500 g/ewe/day of chopped alfalfa hay. The animals were milked twice daily at 7.30 a.m. and 5.30 p.m. and individual milk production was recorded weekly. At each of the two daily milkings, rolled barley (100 g) and one of the two concentrates (125 g) were individually offered. The barley and pelleted concentrate that was offered were completely consumed by all the animals. The CTRL group received a corn-barley based concentrate, while the concentrate offered to the PP group contained 64.8% of dried pomegranate pulp. All the ingredients of the experimental concentrates were pelleted (5 mm diameter) at a temperature ranging from 35–40 °C using a pelleting machine (CMS-IEM, Colognola ai Colli, Verona, Italy). Over a period of 14 days, the animals were gradually adapted to the experimental concentrates, formulated to be isoproteic and according to the nutrient requirement for a ewe weighing 68 kg and producing 0.6 kg of milk at 6.5% of fat [12]. Table 1 reports the ingredients and the chemical composition of the offered feeds. The animal and feeding management that was adopted exactly reflected the common practices used in the commercial sheep farms oriented to dairy production in the Mediterranean regions, where ewes graze all together during the day and receive concentrate supplement during the milking. No practice causing pain, suffering, distress or prolonged damage equivalent or superior to that caused by the insertion of a needle according to the good veterinary practices was followed. Therefore, this study was not regulated by the Directive 2010/63/EU on the protection of animals used for scientific purposes and the approval by the ethic committee was not needed according to the institutional and national guidelines. Nevertheless, the animals were handled by skilled personnel and in accordance with the European legislation on the protection of animals used for scientific purposes (Directive 2010/63/EU).

### 2.3. Sampling and Analyses

#### 2.3.1. Feed

Pasture samples were hand plucked as previously reported by D’Urso et al. [13] before each grazing period and immediately freeze dried. Offered hay, rolled barley, and the experimental concentrates were sampled weekly and stored at −30 °C. The subsamples of feeds, collected weekly, were pooled and analyzed for CP, ether extract (EE), and ash according to the official methods of the Association of Official Analytical Chemists (AOAC) [14], while neutral detergent fiber (NDF), acid detergent fiber (ADF), and acid detergent lignin (ADL) were determined according to Van Soest et al. [15] using heat stable amylase and sodium sulphite, and expressed inclusive of residual ash. The metabolizable energy for lactation was calculated according to Cannas et al. [12].

Total phenolic compounds and total tannins in feeds were analyzed following the procedure originally described by Makkar et al. [16], with modifications as follows. In a 50 mL centrifuge tube, 10 mL acetone 70% (*v*/*v*) was added to 200 mg of finely ground feeds. Samples were vortex-mixed for 1 min and sonicated in a cold-water bath (4 °C) for 15 min. The tubes were centrifuged at 2500× g for 15 min at 4 °C and the supernatant was collected. The residual solid pellet was re-extracted exactly as above using 10 mL methanol 80% (*v*/*v*). The supernatants were combined, and organic solvents were evaporated within 6 min, under vacuum, using a rotary evaporator system (Büchi, R-114, Switzerland) set at 45 °C. The remaining (4 mL) water was mixed with 8 mL methanol and samples were stored at −80°C. For analyses, extracts from pomegranate pomace and pomegranate-containing concentrate were diluted 1:3 with methanol/water (2:1, *v*/*v*). Total phenolic compounds were quantified by mixing: 100 μL sample extract, 900 μL distilled water, 500 μL Folin-Ciocalteu reagent (1 N), and 2.5 mL sodium carbonate 20% (*w*/*v*). The mixture was vortex-mixed, incubated in the dark for 40 min, and centrifuged at 2500× g for 10 min at 4 °C. The absorbance was measured at 725 nm using a double beam UV/VIS spectrophotometer model UV-1601 ( Shimadzu Corporation, Milan, Italy). For the analysis of no-tannin phenolics, sample extracts were first treated with insoluble polyvinylpolypyrrolidone (PVPP) in order to remove tannins. Specifically, 2 mL of sample extract (diluted 1:1 with distilled water) was added to 100 mg PVPP, incubated for 20 min at 4 °C and centrifuged at 2500× *g* for 20 min at 4 °C. The supernatant (200 μL) was mixed with 800 μL of water and the analysis of no-tannin phenols was performed using Folin–Ciocalteu reagent and 20% sodium carbonate, as previously described. Tannins were calculated as the difference between total phenols and total no-tannin phenols. In all the assays, quantification of phenolic compounds was achieved using standard solutions of tannic acid (TA) ranging from 0 to 100 μg/mL. Total phenols and tannins were expressed as g TA equivalents per kg DM.

#### 2.3.2. Milk

Individual milk samples were collected weekly and subdivided into two aliquots. One aliquot was immediately analyzed for fat, protein, casein, and lactose by infrared method (Milkoscan 6000 FT, Foss Electric, Hillerød, Denmark), and total SCC by Fossomatic 5000 (Foss Electric, Hillerød Denmark) and expressed as a linear score (linear score = log2(SCC/12.500)), while the other aliquot was stored at −80 °C pending analyses.

Milk protein fractions were analyzed by capillary zone electrophoresis according to [17] using a Beckman P/ACE MDQ capillary electrophoresis system controlled by 32 Karat software, version 8.0 (Beckman Instruments, Fullerton, CA, USA), equipped with an UV detector set at 214 nm and an uncoated fused silica capillary (57 cm length, 50 μm i.d., 375 μm O.D., slit opening 100 × 800 μm) (Beckman Instruments, Fullerton, CA, USA). The distance between the window and the outlet was 10 cm resulting in an effective capillary length of 47 cm. The sample solutions were injected for 20 s at 0.5 psi. Electrophoresis runs were carried out at 45 °C with a linear voltage gradient from 0 to 25 kV in 3 min, followed by a constant voltage at 25 kV. The sample buffer (pH 8.6 ± 0.1) was 167 mM hydroxymethyl-aminomethane, 42 mM 3-morpholinopropanesulphonic acid, 67 mM ethylenediamine-tetraacetic acid disodium salt dehydrate, 17 mM d,l-dithiothreitol, and 6 M urea and 0.05% (*w*/*w*) hydroxypropylmethylcellulose (MHPC). The run buffer (pH 3.0 ± 0.1) was 0.19 M citric acid, 20 mM sodium citrate, 6 M urea, and 0.05% (*w*/*w*) MHCP. The individual samples were prepared by mixing individual milk and sample buffer (1:2), after 1 h at room temperature, samples were centrifuged at 5000× *g* for 5 min and fat removed. The samples were analyzed without further preparation. Individual proteins were identified by comparison with the electropherograms reported by Clément et al. [18] and the relative concentration determined on the basis of the equation reported by Valenti et al. [17].

Fat from feeds and individual milk samples was extracted with a mixture of chloroform and methanol (2:1, vol/vol) as described by Folch et al. [19] and 25 mg of lipids were converted to FAME by base-catalyzed transesterification [20] using 0.5 mL of sodium methoxide in methanol 0.5 N and 1 mL of hexane. Nonadecanoic acid (19:0) was used as an internal standard. Gas chromatographic analysis was carried out according to Valenti et al. [21] using a Trace Thermo Finningam GC equipped with a flame ionization detector (FID) (ThermoQuest, Milan, Italy) and 100-m high polar fused silica capillary column (25 mm i.d., 0.25 μm, film thickness) SP 24056 (Supelco, Bellefonte, PA, USA). Helium was the carrier gas at a constant flow of 1 mL/min and samples were injected at a 1:80 split ratio. The GC conditions were: 40 °C oven temperature held for 4 min, then increased to 120 °C at 10 °C/min and held for 1 min, then increased up to 180 °C at 5 °C/min and held for 18 min, then increased up to 200 °C at 2 °C/min and held for 15 min, and then increased up to 230 °C at 2 °C/min and held for 19 min. The injector and detector temperatures were 270 °C and 300 °C, respectively. The individual fatty acid peaks were identified by comparison of retention times with those of a known mixture created by mixing a 52 components FAME mix (Nu-Chek Prep. Inc., Elysian, MN, USA) and individual FAME standards (Sigma-Aldrich) run under the same operative conditions. Milk fatty acids were expressed as g/100 g of total fatty acids.

#### 2.3.3. Total Antioxidant Capacity of Feeds and Milk

The antioxidant capacity of feeds and milk was determined using the oxygen radical absorbance capacity (ORAC) method based on the fluorescence decay rate of a probe in the presence of a radical oxygen species as compared with that of a reference standard, Trolox (6-hydroxy-2,5,7,8-tetramethylchroman-2-carboxylic acid, Sigma-Aldrich, Steinheim, Germany). The hydrophilic and lipophilic fractions of the milk samples (5 g) or feed sample (5 g of finely powdered feed) were extracted according to Prior et al. [22] to evaluate the lipophilic (L-ORAC) and hydrophilic (H-ORAC) ORAC. Measurements of L-ORAC and H-ORAC in the samples were performed separately, and the total antioxidant capacity (TAC) was calculated by summing the L-ORAC and H-ORAC values, as described by Wu et al. [23]. The ORAC assays were carried out on a FLUOstar OPTIMA microplate fluorescence reader (BMG LABTECH, Offenburg, Germany) set at an excitation wavelength of 485 nm and an emission wavelength of 520 nm. The procedure was based on the method of Zulueta et al. [24] with slight modifications. Briefly, 2,20-azobis (2-methylpropionamide) dihydrochloride (AAPH; Sigma-Aldrich) was used as peroxyl radical generator, Trolox was used as a reference antioxidant standard, and fluorescein was used as a fluorescent probe. A 100 µL volume of diluted sample, blank or Trolox calibration solution (10–80 µmol) was mixed with 1 mL of fluorescein (80 nM). Then, 200 µL of each mixture was placed in a well of the microplate. The microplate was placed in the reader and pre-incubated for 20 min at 37 °C. To each well, 60 µL of AAPH was automatically added to initiate the reaction and the fluorescence was recorded every 1.9 min. All the reaction mixtures were prepared in duplicate, and at least three independent assays were performed for each sample. The final ORAC values were calculated by using a linear regression equation (Y = a + bX) to describe the relationship between the Trolox concentration (Y, 10–80 µM Trolox) and the net area under the fluorescence decay curve (X). The data were expressed as µmol Trolox equivalents (TE) per g of sample by applying the following formula:

ORAC (μmol TE) = [CTrolox (AUCSample − AUCBlank) k] / (AUCTrolox − AUCBlank)

where, CTrolox is the concentration of Trolox, k is the sample dilution factor, and AUC is the area below the fluorescence decay curve of the sample, the blank, and Trolox, respectively, calculated by applying the following formula [25]

AUC = 0.5 + f1 / f0 + … fi / f0,

where, f1 is the initial fluorescence reading at t = 0 min and fi is the fluorescence reading at time = i. The net AUC for each sample was obtained by subtracting the AUC of the corresponding blank from that of the sample.

### 2.4. Statistical Analysis

Individual data on milk yield, chemical composition, protein profile, antioxidant capacity, and fatty acid composition were statistically analyzed using the general linear model procedure for repeated measures, to text the effect of dietary treatment, time of sampling and their interaction. The individual animal was included as a random factor. The pre-treatment data of each parameter was used as a covariate. When the covariate was not significant (*p* > 0.05) it was excluded from the model. Tukey’s adjustment was used for the multiple comparisons of the means. Significance was declared when *p* ≤ 0.05. The analyses were performed using the statistical software Minitab, version 16 (Minitab, Inc., State College, PA, USA).

## 3. Results

Table 2 reports data on milk production, chemical composition, and protein profile of the milk. Dietary treatment did not affect milk yield, percentage of fat, protein and lactose, and the concentration of urea. Somatic cell count, expressed as linear score, was greater in the PP than the CTRL group (*p* = 0.031). With respect to the milk protein profile, *α*_s1_-casein *(α*_s1_-CN) and *β*-casein (*β*-CN) were the most abundant proteins in the milk for both of the groups, representing more than 30% each, followed by *β*-lactoglobulin (*β*-Lg), *α*_s2_-casein (*α*_s2_-CN), *κappa*-casein (*κ*-CN), and *α*-lactalbumin (*α*-Lac). The percentage of *α*s1-CN tended to be greater in the PP than in the CTRL (*p* = 0.056), while *β*-CN and total casein were greater in the CTRL than the PP group (*p* = 0.048 and *p* = 0.002, respectively).

Table 3 reports the effect of dietary treatment on milk fatty acid composition. The percentages of 14:0, 16:0, *cis*-9 14:1, and *cis*-9 16:1 were greater in the CTRL than in the PP milk. In contrast, the percentages of *trans*-9 18:1, *trans*-11 18:1 (VA), *cis*-6 18:1, *cis*-9 *trans*-11 18:2 (RA), and *cis*-9 *cis*-12 *cis*-15 18:3 (ALA) were greater in the PP than the CTRL group. Punicic acid was detected only in the milk of PP ewes, representing 0.19% of total milk fatty acids. Regarding the sums of fatty acids, the CTRL milk showed a greater percentage of SFA and a lower proportion of PUFA. Moreover, the CTRL milk had a lower percentage of total PUFA n-3. The dietary treatment did not affect the sum of MUFA, odd and branched chain fatty acids (OBCFA), and PUFA n-6.

**Figure 1:**
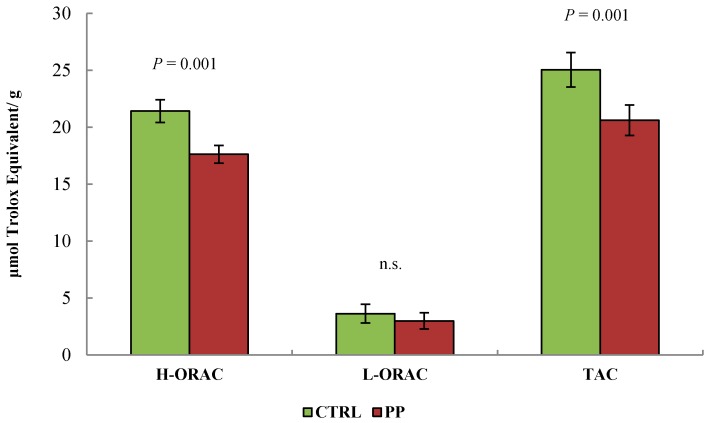
Effect of the inclusion of pomegranate pulp in the diet of lactating ewes on the antioxidant capacity of milk expressed as (μM Trolox Equivalent/g). CTRL = control concentrate, PP = concentrate containing 64% pomegranate pulp, H-ORAC = antioxidant capacity of milk hydrophilic fraction, L-ORAC = antioxidant capacity of milk lipophilic fraction, TAC = total antioxidant capacity of milk (H-ORAC + L-ORAC).

Figure 1 reports the effect of dietary treatment on the milk antioxidant capacity. The results of the ORAC assay showed that the H-ORAC accounted for most of the TAC in milk as compared with the L-ORAC. Dietary treatment affected the TAC, with higher values found in the CTRL group as compared with the PP (*p* = 0.006). These differences in the TAC values were due to the effect of the dietary treatment on the antioxidant capacity measured in the H-ORAC, which were higher for the CTRL group as compared with the PP group (*p* = 0.002). Conversely, no difference between treatments was observed for L-ORAC.

## 4. Discussion

In the present study, 648 g/kg DM dried pomegranate pulp was included in a pelleted feed given to grazing ewes in order to evaluate the effect of dietary pomegranate by-product on milk yield and quality traits during late lactation. According to Cannas et al. [12], it was estimated that that the ingestion of the individual ewe was about 2.3 kg, and therefore the pomegranate pulp represented about 7% of total dry matter intake (DMI). The chemical composition of PP used in the present paper was in the range reported for similar pomegranate by-products [10,11,26]. The crude fat of PP mostly arises from the seed oil, and therefore, it is not surprising that PA was the most abundant fatty acid in the fat of PP. The total phenolic compounds of pomegranate pulp were represented mainly by tannins, which is consistent with previous study [27].

Tannins are water-soluble phenols characterized by their potential to create complexes with the proteins and, to a lesser extent, with metals and ions [28]. Due to this chemical property, dietary tannins can have both adverse and beneficial effects on ruminants, depending on factors such as dose, chemical composition, and characteristics of the basal diet [29]. Regarding dairy ruminants, tannins can increase milk protein percentage and reduce urea content [30] by favoring the outflow of protein nitrogen from the rumen [31], which is one of the limiting factors for milk production.

Controversial results are reported on the effect of dietary pomegranate by-products on milk yield and composition. Kotsampasi et al. [10] did not observe a variation of DMI, milk yield, and gross composition when 75 or 150 g/kg of pomegranate silage were included in the diet of lactating cows. A similar result was observed by Modaresi et al. [8] in lactating goats with the inclusion of 60 or 120 g/kg of pomegranate seed pulp. In contrast, Shaani et al. [7] found that when 8% of ensiled PP replaced corn grain in a total mixed ration it reduced DMI and milk yield in cows, while lambs refused to consume a TMR containing more than 20% of pomegranate by-product [26]. In the present trial, PP concentrate was completely consumed, despite the fact that the level of PP inclusion was greater than values reported in the literature and the tannins content was 61 g/kg DM. Moreover, no effects on milk yield, fat and protein percentage, and urea content were observed.

This inconsistency among the studies could be due to several factors. In their review, Jeronimo et al. [32] stated that the species used in the different trials may show a variable sensitivity to the astringent sensation induced by the type and quantity of tannins in the feeds. Min et al. [33] indicated that a dose of dietary tannins greater than 50 g/kg DM can reduce the voluntary feed intake and animal performance, however, this limit should be applied to the total ingested diet and not to a specific component. Finally, the potential of tannins to improve animal performance seems to depend also on the physiological status of the animals. Waghorn [34] reported that the surplus of protein escaping the rumen, generated by the protective action of tannins, is better exploited by ewes which have greater protein requirements as compared with less productive animals. Interestingly, we observed that the dietary treatment affected individual milk proteins percentage. In particular, *β*-CN and total casein were higher in the CTRL group as compared with the PP group.

The availability of essential amino acids (EAA) represents the most limiting factor for the endogenous biosynthesis of milk protein in the mammary gland. According to Orlandi et al. [35], feeding tannins to ruminants increased the flux of EAA through the abomasum and to the duodenum [35] with positive effects on milk protein yield. Surprisingly, our results contradict these observations. This is the first study reporting the effect of dietary pomegranate by-products on milk protein profile, and the lack of knowledge on the effect of dietary polyphenols on individual milk proteins does not allow a comparison with the literature. However, in a similar study, dietary chestnut hydrolysable tannins increased protein yield and casein index without affecting individual caseins in the milk from ewes during mid-lactation [30]. A possible explanation for the lower percentage of *β*-CN in the PP group could be the greater somatic cell count, expressed as LS, observed in PP milk. It is known that LS is positively correlated with the activity of plasmin, a protease that degrades *β*-CN into γ-CN. Although the effect of plasmin is evident at values of LS greater than those recorded for both the groups in the present study, it cannot be excluded as a possible cause of the lower *β*-CN and total casein in PP milk.

The antioxidant capacity of fresh pomegranate has been associated with the presence of tannins, anthocyanins, and other phenolic compounds in the different portions of the fruit [2]. The potential of polyphenols to act as dietary antioxidants with the aim of improving the antioxidant properties of ruminant products has been investigated [5], but it remains a controversial issue. In the present study, the ORAC test was used to evaluate the free radical-scavenging capacity of milk and it revealed that TAC was greater in the CTRL than in the PP milk. Specifically, this difference was due to a greater H-ORAC of CTRL milk, while the L-ORAC was comparable between the groups.

Both direct and indirect antioxidant mechanisms of phenolic compounds in vivo have been suggested. A direct effect is linked to the possibility that these compounds are absorbed in the gut in order to reach the tissues. It has been reported that phenols with a low molecular weight can be absorbed and transferred to the blood stream [5]. Pomegranate phenols are mainly represented by hydrolysable tannins [3], which, differently from the condensed tannins, can be degraded by rumen microbes. Therefore, it is likely that small products of degradation of pomegranate tannins can be absorbed. Consistently, Kotsampasi et al. [11] showed that the total phenols in lamb meat were linearly correlated to the level of pomegranate silage inclusion in the diet of growing lambs. However, in the same paper, though the meat antioxidant capacity was greater in the groups receiving the pomegranate silage, it was not linearly correlated to the presence of phenols in meat, suggesting that the accumulation of antioxidant molecules is not the only mechanism responsible for the antioxidant capacity of the animal products.

Other authors found that the improvement in antioxidant capacity of different tissues was not related to a direct transfer of phenolic compounds from the diet [36], suggesting that the antioxidant activity of dietary phenolic compounds could be indirectly mediated by an effect on the overall oxidative status of the animal organism. The balance between pro-oxidant and antioxidant components is an important factor affecting the antioxidant properties of animal products. In particular, the oxidation of susceptible compounds, such as PUFA, is prevented by endogenous defense systems and antioxidants of direct and indirect derivation from the diet. Zulueta et al. [24] reported that the major peroxyl radical scavengers in milk are the caseins, among which *β*-CN is the most prominent [37]. On the one hand, this could partially explain the higher H-ORAC values found in CTRL milk, which had a greater percentage of *β*-CN and total casein as compared with PP. On the other hand, PP milk had a higher percentage of both total PUFA and PUFA n-3. Therefore, it may also be supposed that, as compared with CTRL, anti-oxidants could have been consumed more in the PP milk, in order to counteract the higher susceptibility of PUFA to oxidation.

With respect to milk fatty acids, PP milk had a greater percentage of total PUFA and PUFA n-3 and a lower proportion of 14:0, 16:0, and total SFA. Similar results were reported when pomegranate seeds oil [9] or seed pulp [8] were added to the diet of goats. The reduction of total and individual SFA in milk and meat is a common result when dietary sources of PUFA are included in the diet of ruminants [38]. A high quantity of 14:0 and 16:0 in foods is considered harmful for human health because they increase the atherogenic and the thrombogenic index. Therefore, the inclusion of PP in ruminant diet could be a valid strategy to improve the healthiness of milk by reducing these undesirable FA in favor of healthy FA such as PUFA and PUFA n-3.

In addition, PA was detected only in the PP milk. Punicic acid is a double-conjugated trienoic fatty acid (*cis*-9 *trans*-11 *cis*-13 18:3) that represents the main fatty acid in the oil of pomegranate seeds [39]. Its therapeutic and preventive properties against obesity, diabetes, and cardiovascular disease have been recently reviewed [4]. In the present study, PA accounted for 60% of the total fatty acids in the PP concentrate, while it represented only 0.2% of milk fatty acids. Considering that PA in milk exclusively arises from the ingested diet, it is likely that PA was extensively biohydrogenated in the rumen of PP ewes. The ruminal biohydrogenation pathway of PA has not yet been reported. However, it is plausible that, similar to the biohydrogenation of linoleic acid (LA) and ALA [38], VA and RA are intermediates of PA biohydrogenation. In line with this hypothesis, the proportion of VA and RA was higher in the PP milk.

There are many reports that tannins can protect dietary PUFA from the biohydrogenation [5,40]. On the one hand, our results could lead to the conclusion that the protective effect of tannins on the dietary punicic acid occurred minimally. On the other hand, looking at the fatty acid composition of the experimental concentrates, an effect of tannins on the biohydrogenation cannot be excluded. The CTRL concentrate was richer in LA and ALA as compared with PP, and therefore a greater percentage of these two fatty acids would have been expected in the CTRL milk. However, ALA was greater in PP milk, while LA was comparable between the groups. According to Chilliard et al. [38], LA and ALA represent the elective substrates for biohydrogenation. Thus, it could be supposed that the biohydrogenation of these compounds occurred normally in the rumen of CTRL ewes, while it was reduced in the PP group.

## 5. Conclusions

The results reported in the present study indicated that the inclusion of pomegranate pulp in the diet of grazing ewes did not affect milk yield and gross composition during late lactation. However, dietary pomegranate pulp enriched the milk with healthy fatty acids such as RA, VA, and punicic acid. These findings suggest that recycling pomegranate pulp as feedstuffs to reduce conventional concentrates could reduce both the feeding costs during a less profitable phase, such as the late lactation, and the feed-to-food competition in livestock production. Our results could be of particular interest in some areas, such as the Mediterranean, where lambing seasons are managed in order to concentrate most of the lactation when pasture is abundant, while late lactation occurs when summer approaches and pasture availability and quality decline, and therefore concentrate feeds and hay are necessary to meet nutrient requirements. These conditions contribute to reduce the quality of milk produced during late lactation, and the administration of dietary pomegranate pulp could represent a valid strategy to maintain a high level of milk quality. Further study should be done to assess the effect of dietary pomegranate pulp during a different phase of lactation. Finally, it would be of interest to clarify the role of the punicic acid and of tannins in the mechanism that led to an increased percentage of RA and VA in the milk of the PP group.

## Figures and Tables

**Table 1:** Ingredients and chemical and fatty acid composition of pomegranate pulp, basal diet (pasture, hay, and barley), and experimental concentrates fed to the lactating ewes.

Item	Pomegranate Pulp	Pasture	Hay	Barley	Concentrate ^1^	
					CTRL	PP
Ingredients (g/kg dry matter)						
Wheat bran					205.6	71.4
Corn					345.0	40.1
Barley					336.0	32.7
Soybean meal					35.5	130.4
Pomegranate pulp					-	648.4
Molasses					51.9	51.9
Mineral mix					25.9	25.6
Chemical composition (g/kg dry matter)						
Dry matter	911.6	22.3	87.6	88.9	85.6	85.3
Crude protein	68.8	135.4	156.2	118.6	140.6	139.8
Crude fat	44.3	26.0	15.9	34.4	30.5	38.9
Neutral detergent fiber	314.0	485.1	443.3	269.3	221.0	237.1
Acid detergent fiber	227.8	318.8	348.9	102.5	100.9	159.6
Acid detergent lignin	68.8	35.9	70.7	24.4	20.3	39.4
Ash	36.4	93.3	98.7	26.8	61.2	67.4
ME ^2^	10.7	10.4	9.52	13.47	10.9	12.2
Total phenols ^3^	95.3	2.01	8.22	0.59	1.25	61.4
Total tannins ^3^	93.4	-	2.48	0.03	-	60.9
Fatty acids (g/100 g total fatty acids)						
14:0	0.06	0.42	0.78	0.33	0.14	0.07
16:0	3.54	13.6	20.7	20.2	14.2	5.80
18:0	1.92	1.43	3.90	2.49	2.05	2.05
*cis*-9 18:1	5.43	3.25	4.85	14.1	22.0	7.84
*cis*-9 *cis*-12 18:2	5.87	15.8	16.6	55.0	50.2	14.1
*cis*-9 *cis*-12 *cis*-15 18:3	0.29	56.9	35.9	4.17	2.73	1.11
*cis*-9 *trans*-11 *cis*-13 18:3	71.2	-	-	-	-	57.7

^1^ Concentrate: CTRL = control concentrate, PP = concentrate containing 64% pomegranate pulp; ^2^ ME = metabolizable energy expressed as MJ/kg dry matter; ^3^ Expressed as g of tannic acid equivalent/kg dry matter.

**Table 2:** Effect of the inclusion of pomegranate pulp in the diet of lactating ewes on milk production and composition.

Item	Concentrate ^1^ (Diet)		SEM ^2^	*p* Value		
	CTRL	PP		Diet	Day	Diet × Day
Milk yield (g/day)	332.2	339.7	19.9	0.910	0.252	0.025
Fat (%)	8.29	8.30	0.110	0.961	0.630	0.366
Protein (%)	6.53	6.45	0.069	0.521	0.064	0.764
Lactose (%)	4.29	4.21	0.048	0.382	0.725	0.604
Urea (mg/mL)	55.0	54.7	1.35	0.931	0.071	0.644
Linear score	3.40	4.30	0.227	0.031	0.609	0.406
Protein fractions (% total protein)						
*α*-lactalbumin	2.19	2.30	0.076	0.441	0.031	0.447
*β*-lactoglobulin	14.2	14.6	0.167	0.277	0.063	0.142
*α*_s2_-casein	6.49	6.71	0.177	0.467	0.118	0.557
*α*_s1_-casein	33.9	35.3	0.368	0.056	0.590	0.542
*κ*-casein	6.02	5.92	0.179	0.801	0.469	0.280
*β*-casein	33.6	31.3	0.592	0.048	0.209	0.477
Whey protein	16.4	16.9	0.204	0.234	0.030	0.119
Total casein	80.3	79.0	0.321	0.002	0.002	0.032

^1^ Concentrate: CTRL= control concentrate, PP = concentrate containing 64% pomegranate pulp; ^2^ SEM = standard error of mean.

**Table 3:** Effect of the inclusion of pomegranate pulp in the diet of lactating ewes on milk fatty acids expressed as g/100 g of total fatty acids.

Item	Concentrate ^1^ (Diet)		SEM ^2^	*p* Value		
	CTRL	PP		Diet	Day	Diet × Day
4:0	2.08	2.19	0.066	0.328	<0.001	0.604
6:0	1.74	1.83	0.048	0.252	0.001	0.742
8:0	1.66	1.77	0.047	0.190	0.050	0.953
10:0	5.34	5.53	0.168	0.581	0.272	0.993
12:0	3.07	3.09	0.098	0.925	0.171	0.925
*cis*-9 12:1	0.15	0.14	0.005	0.542	0.037	0.970
14:0	9.97	9.22	0.201	0.056	0.163	0.866
*cis*-9 14:1	0.26	0.21	0.011	0.027	0.396	0.985
*iso* 15:0	0.38	0.37	0.006	0.474	0.531	0.317
*anteiso* 15:0	0.66	0.63	0.012	0.274	0.521	0.404
15:0	1.43	1.44	0.015	0.911	0.000	0.759
*iso* 16:0	0.36	0.37	0.008	0.938	0.504	0.711
16:0	28.8	26.4	0.383	0.001	0.132	0.627
*cis*-9 16:1	1.26	1.04	0.037	0.003	0.618	0.859
*iso* 17:0	0.59	0.62	0.011	0.193	0.470	0.589
*anteiso* 17:0	0.64	0.67	0.016	0.276	0.097	0.237
17:0	0.87	0.92	0.016	0.090	0.025	0.964
18:0	8.86	9.20	0.213	0.427	0.104	0.991
*trans*-*6+7+8* 18:1	0.14	0.17	0.007	0.106	0.984	0.835
*trans*-9 18:1	0.25	0.32	0.007	<0.001	0.592	0.480
*trans*-10 18:1	0.22	0.23	0.007	0.509	0.678	0.756
*trans*-11 18:1	1.04	1.45	0.006	<0.001	0.974	0.987
*cis*-6 18:1	0.71	0.85	0.029	0.014	0.115	0.788
*cis*-9 18:1	18.8	18.8	0.361	0.976	0.013	0.921
*cis*-11 18:1	0.38	0.41	0.014	0.342	0.021	0.770
*cis*-12 18:1	0.22	0.25	0.010	0.118	0.521	0.999
*cis*-9 *trans*-11 18:2	0.69	1.28	0.051	<0.001	0.999	0.779
*cis*-9 *cis*-12 18:2	2.49	2.70	0.060	0.078	0.432	0.732
*cis*-6 *cis*-9 *cis*-12 18:3	0.48	0.42	0.002	0.218	0.457	0.876
*cis*-9 *cis*-12 *cis*-15 18:3	1.41	1.60	0.044	0.024	0.132	0.711
*cis*-9 *trans*-11 *cis*-13 18:3	n.d.	0.19	0.018	−	−	−
20:0	0.36	0.36	0.008	0.917	0.123	0.902
*cis*-11 20:1	0.58	0.64	0.002	0.108	0.803	0.849
20:3 n-6	0.03	0.03	0.002	0.622	0.698	0.809
20:4 n-6	0.23	0.22	0.006	0.695	0.371	0.842
20:5 n-3	0.10	0.10	0.003	0.561	0.121	0.689
22:2	0.03	0.04	0.002	0.224	0.004	0.924
22:4 n-6	0.04	0.04	0.003	0.253	0.457	0.705
22:5 n-6	0.10	0.09	0.003	0.277	0.235	0.821
22:6 n-3	0.19	0.20	0.005	0.288	0.107	0.789
24:0	0.12	0.11	0.011	0.818	0.675	0.524
Σ SFA ^3^	62.1	59.7	0.563	0.029	0.206	0.757
Σ MUFA ^4^	23.5	23.9	0.375	0.566	0.023	0.885
Σ PUFA ^5^	5.40	6.48	0.159	<0.001	0.595	0.783
Σ PUFA n-3	1.70	1.90	0.047	0.025	0.240	0.729
Σ PUFA n-6	2.96	3.16	0.067	0.151	0.463	0.815
Σ OBCFA ^6^	4.94	5.02	0.061	0.503	0.264	0.553

^1^ Concentrate: CTRL= control concentrate, PP = concentrate containing 64% pomegranate pulp; ^2^ SEM = standard error of mean; ^3^ SFA = sum of saturated fatty acids; ^4^ MUFA = sum of monounsaturated fatty acids; ^5^ PUFA = sum of polyunsaturated fatty acids; ^6^ OBCFA = sum of odd and branched chain fatty acids.
